# Open Treatment of Abdominal Aortic Aneurysm in the Endovascular Era

**DOI:** 10.3390/jcm11113050

**Published:** 2022-05-28

**Authors:** Abdulhakim Ibrahim, Miroslav Dimitrov Yordanov, Mohammad Hasso, Benjamin Heine, Alexander Oberhuber

**Affiliations:** Department of Vascular and Endovascular Surgery, University Hospital Muenster, 48149 Muenster, Germany; miroslavdimitrov.yordanov@ukmuenster.de (M.D.Y.); m_hass12@uni-muenster.de (M.H.); benjamin.heine@ukmuenster.de (B.H.); alexander.oberhuber@ukmuenster.de (A.O.)

**Keywords:** aortic aneurysm, open aortic repair, morbidity, mortality

## Abstract

The aim of the study was to analyse outcomes and determine the early and late complications in patients after open surgical treatment of AAA in the endovascular era. Two hundred and fourteen patients between January 2012 and December 2021 with open repair in primary infrarenal and juxtarena aneurysm in elective setting were included in the study. Pre-, intra-, and postoperative clinical data were statistically analysed. The mean age of the 214 patients was 65.5 ± 9.3 years. The mean follow-up was 22.1 ± 2.1 months. Men represented the majority of the studied group (85.5%). The mean aortic diameter was 58.2 ± 13.4. The median ICU (days) stay was 5 ± 4.9 days for infrarenal aneurysm and 6 ± 6.1 days for juxtarenal aneurysm. Four patients died within 30 days, giving an in-hospital mortality rate of 1.9%. In multivariate logistic regression, COPD (*p* = 0.015) was the only predictor significantly associated with the mortality. A comparison of survival and reintervention using a Kaplan–Meier curve showed no significant difference between the groups in terms of risk stratification and the groups with juxtarenal versus infrarenal aneurysms. In conclusion, open aneurysm repair is in the era of endovascular aneurysm repair, being safe and effective, especially when performed in specialised high-volume centres with large expertise.

## 1. Introduction

Abdominal aortic aneurysm (AAA) is a pathological dilatation of the aorta, the diameter of which is 50% larger than normal [1]. The goal of aortic aneurysm repair is to prevent rupture. Open repair of AAA is associated with significantly high morbidity and mortality. A 30-day mortality rate of 5.2% (*n* = 39,966) for elective cases during the period from 2001 through 2008 was reported for AAA [2]. The most common complications after elective open surgical repair (OSR) are cardiac and respiratory events and wound infections, which occur perioperatively in 10.2%, 7%, and 3.5% of patients after surgery, respectively [3]. An analysis of registry data (>35,000 patients) from the German Society for Vascular Surgery and Vascular Medicine (DGG) revealed a mortality of elective AAA therapy of around 3.6% OSR [4]. The aim of our study was to determine the type, frequency, and early and late complications in patients after open surgical repair of abdominal aortic aneurysms in the endovascular era and to analyse the mortality and reintervention rates according to surgical risk and aneurysm type (juxta- vs. infrarenal).

## 2. Materials and Methods

### 2.1. Study Design and Patient Selection

This was a single-centre, retrospective, observational study on a total of 214 consecutive patients who underwent open aortic repair between January 2012 and December 2021. Patient demographics, comorbidities, and intraoperative data, as well as pre- and postoperative computed tomographic angiogram (CTA) findings, vital parameters, and complications were analysed.

### 2.2. Data Collection and Definitions

All data were collected from our digital medical documentation. Wound infection was defined as clinical signs of local wound inflammation and positive culture from this site. Kidney failure was defined as postoperative serum creatinine increase greater than 2 mg/dL from baseline or new dialysis in the 30-day post-operative period, as defined by NSQIP (National Surgical Quality Improvement Program). Lower limb ischemia was defined as acute or critical ischemia, which required surgical intervention. A pulmonary complication was defined as pneumonia, failure to wean from mechanical ventilation within 48 h, re-intubation, or pulmonary embolism. Ileus was defined by postoperative clinical and radiological findings. Urinary tract infection was considered as positive urine bacterial culture and leukocyte count. Bleeding (re-operation) and bowel ischemia were defined as clinical, radiological, and laboratory signs requiring surgical intervention. Patients were stratified according to the surgical risk using the Medicare Aneurysm Scoring System [5]. This scale was developed as a prediction tool for perioperative death in patients treated for AAA. Variables in this model include age, sex, congestive heart failure (CHF), chronic kidney disease (CKD), peripheral arterial disease (PAD), and cerebrovascular disease (CVD). The division of low-, moderate-, and high-risk corresponds to score ranges of <3 (low), 3 to 11 (moderate), and >11 (high) (Table 1).

Primary outcomes were 30-day mortality, 30-day reinterventions, and access-related complications. Secondary outcomes were mortality, aortic rupture, and late reinterventions.

### 2.3. Inclusion and Exclusion Criteria

In our study, only patients with open repair of infrarenal and juxtarenal aneurysm in elective setting were included. All patients who underwent an open treatment with supra- or intrarenal anastomosis and patients for another diagnosis (aortoenteric/aortocaval fistula, Leriche syndrome, postdissection aneurysm, persistent endoleak with sac enlargement despite endovascular treatment, aortitis and prosthesis infection) were excluded from our study cohort. Moreover, all elective endovascular repairs (EVAR) were excluded.

### 2.4. Statistical Analysis

Continuous variables are expressed as mean ± standard deviation for parametric data and median with interquartile range for non-parametric data, whereas dichotomous variables are presented as crude numbers and percentages. A multivariable logistic regression analysis was used to elucidate the independent predictors of in-hospital mortality. We used the univariate analysis for all possible risk factors and tested only the significant variables in a multivariate regression analysis. A Kaplan–Meier curve was used to compare survival and reintervention. The differences between groups were compared using the Mantel–Cox log-rank test. A *p*-value of <0.05 was considered statistically significant. All statistical analyses were performed using SPSS Statistics for Windows version 26.0 (IBM Corp., Armonk, NY, USA).

## 3. Results

A total of 502 patients underwent open aortic repair between January 2012 and December 2021. Only patients with open repair in primary infrarenal and juxtarenal aneurysm in elective setting were included in the study (*n* = 214). In all patients, the proximal anastomosis was situated below the renal arteries. On the basis of the exclusion criteria, 288 patients were excluded due to aortoenteric fistula with aortic prosthesis infection (*n* = 49), Leriche syndrome (*n* = 27), postdissection aneurysm (*n* = 29), persistent endoleak after endovascular treatment (*n* = 22), aortitis (*n* = 2), thoracal and thoracoabdominal aneurysm (*n* = 112), and ruptured aortic aneurysm (*n* = 47).

The mean age was 65.5 ± 9.3 years. One hundred and forty-seven patients (68.7%) were younger than 70 years, and two hundred and three (94.9%) younger than 80 years. Men represented the majority of the studied group (*n* = 183, 85.5%). The mean aortic diameter was 58.2 ± 13.4 mm. Mean follow up time was 22.1 ± 2.1 months. Hypertension (*n* = 129, 60%), coronary artery disease (*n* = 68, 31.8%), and being a current/previous smoker (*n* = 71, 33.2%) were the most common comorbidities. Patients often received the following medications: betablocker (*n* = 93, 43.5%), statin (*n* = 97, 45.3%), and aspirin (*n* = 107, 50%) (Table 2).

The patients were divided into three groups according to the surgical risk using the Medicare Aneurysm Scoring System. Most patients were in the low-risk group (*n* = 134, 62.6%), 61 patients (28.5%) were in the moderate-risk group, and only 19 patients (8.9%) were in the high-risk group (Table 3). In most patients, a transperitoneal approach (*n* = 182, 85%) was used. Eighty patients (37.3%) needed a temporary suprarenal clamping of at least one renal artery. After performing the proximal anastomosis, the prosthesis was clamped out infrarenal. The mean clamping time was 26.1 ± 2.8 min. The IMA (inferior mesenteric artery) was reimplanted only in 14 patients (6.5%). Indication was occlusion of the hypogastric arteries or inadequate back bleeding. The mean operation duration was (189.5 min ± 78.7). The median ICU stay was 5.0 ± 4.9 days for infrarenal aneurysm and 6.0 ± 6.1 days for juxtarenal aneurysm. The median in-hospital stay for infrarenal aneurysm was 10.0 ± 12.4 days, and for juxtarenal aneurysm, 11.0 ± 7.7 days (Table 3).

### 3.1. Complications and Reinterventions after OSR

Fifteen patients required reintervention in the first 30 days after OSR. Postoperative wound infection, including burst abdomen, were registered in 13 patients (6.1%), with only 6 patients requiring reintervention. Postoperatively, ileus developed in 12 patients, and only 2 patients were reintervened for this purpose. Bowel ischemia was registered in four patients. All four patients underwent bowel resection. One of them died due to the attendant complications. Other complications included lower limb ischemia (*n* = 6, 2.6%), pneumonia (*n* = 12, 5.6%), myocardial infarction (*n* = 3, 1.4%), urinary tract infection (*n* = 3, 4.2%), and bleeding (*n* = 3, 1.4%). The most common late complication after OSR was incisional hernia (*n* = 17, 7.5%). Kidney failure was registered in five patients (2.3%), and kidney failure requiring dialysis was not reported in any patient. Other complications included anastomosis aneurysm (*n* = 2, 0.9%), prosthesis infection (*n* = 4, 1.9%), and vascular prosthesis obstruction (*n* = 1, 0.5%) (Table 4).

### 3.2. Mortality

Of the 214 patients who had elective open abdominal repair for aortic aneurysm, four died within 30 days, giving a surgical mortality rate of 1.9%. One patient had complicated sigmoid ischemia as a postoperative complication, and the three remaining patients died as a result of cardiovascular events. In the follow-up, eight further deaths were registered. Three patients died from malignancy, and two patients from cardiovascular events. The cause of death in the remaining three patients remained unclear. On the basis of the univariable analysis, different mortality predictors such as COPD, high risk factor of the patient, and transfusion of PRBS were determined. In the multivariate logistic regression, the COPD (*p* = 0.015) remained the only variable significantly associated with mortality (Table 5).

**Figure 1:**
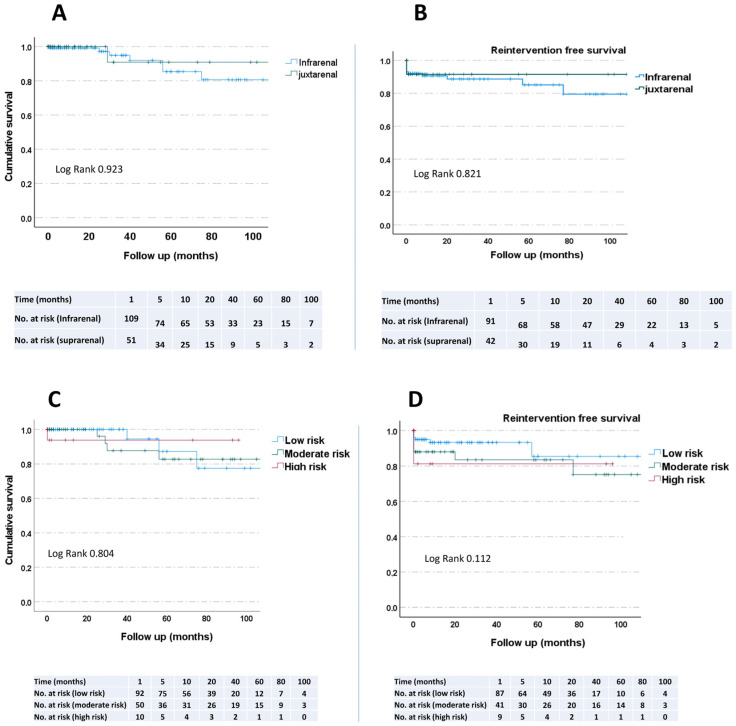
Kaplan–Meier survival and reintervention estimates showed no significant difference between the groups. (**A**,**B**) Patients stratified into two groups: infrarenal aneurysm (*n* = 137) and juxtrarenal aneurysm (*n* = 77). (**C**,**D**) Patients were stratified into three groups according to the surgical risk (Medicare Aneurysm Scoring System): low risk (*n* = 137), moderate risk (*n* = 61), and high risk (*n* = 19).

Patients with different degrees of aortic pathology and different increased risk for surgery could show different late mortality and reintervention rate, and thus we evaluated our patients according to Kaplan–Meier survival/reintervention estimate curves. At late follow-up for patients undergoing elective open abdominal aortic aneurysm repair, a comparison of survival and juxta- versus infrarenal aneurysm gave a *p*-value of 0.923 (Figure 1A). The reintervention also did not differ between the two aneurysm types (Figure 1B). A comparison of survival and reintervention between the patients in Kaplan–Meier estimate curves according to the risk stratification also showed no significant difference between the groups (survival: *p* = 0.80, reintervention: *p* = 0.11) (Figure 1).

## 4. Discussion

There have been 72 years since the first successful abdominal aneurysm repair by Charls Dubost (1951) with an aortic homograft, which is considered to be the beginning of the modern era of the aneurysm repair [6]. In the following decades, the introduction of aortic prostheses along with the constant improvements of the surgical and anaesthesiologic care led to the standardisation of the open surgical aneurysm repair with mortality rates of less than 5%.

In 1989, Volodos introduced the endovascular aneurysm repair [7]. With the improvement of the endovascular materials and diagnostic techniques, this novel approach became rapidly the preferred method of treatment of AAA, due to its low mortality and complication rates.

It is well known that the preoperative clinical examination and risk factors estimation plays a crucial role in the decision making and choice of the individual therapeutic approach for each patient. According to the literature, the cardiovascular and pulmonary diseases are the leading cause of early and late death after aortic aneurysm operations. Among these high-risk patients, EVAR is associated with threefold reduction in perioperative mortality, compared with propensity-matched patients undergoing elective OSR [6,7,8]. For patients with COPD and chronic renal failure, the outcome after EVAR is better than by OSR [9,10]. It is not surprising that the EVAR rapidly became the first-choice therapy, and nowadays nearly 80% of the patients in the USA are treated endovascularly.

The OSR is a preferred method of treatment by patients unsuitable for EVAR due to anatomic reasons such as short or angulated aneurysm neck (hostile neck), large accessory renal arteries, excessive thrombus, or unsuitable access arteries. The patients with complex aneurysm morphology can still be operated endovascularly with a custom-made stent graft, but the long production and delivery time could possibly increase the risk of aortic rupture. If the perioperative risk in not significantly increased, they can safely undergo an OSR.

In patients with low to intermediate perioperative risk, OSR is as safe as EVAR [11]. However, the studies demonstrated a higher reintervention rate after EVAR than those undergoing OSR. Operative complications, health-related quality of life, and sexual dysfunction were generally comparable between EVAR and OSR [12]. These results are consistent with the observations in the EVAR1 study conducted in the UK with follow-up of 15 years. Total and aneurysm-related mortality were lower in patients who received EVAR in the first 6 months, but increased after 6 months follow-up, leading to a significantly higher rate after 8 years follow-up in EVAR than in those who received OR. After the first 6 months, the increased aneurysm-related deaths in the EVAR group were predominantly from secondary sac rupture. The rate of reintervention was higher in the EVAR group at all time periods. On the basis of these conclusions, we could suggest that the OSR is a more durable treatment option for young patients with relatively low perioperative risk.

The repair of juxtarenal aortic aneurysms (JAA) still raises a large number of debates. In many cases, due to the complex aneurysm morphology with inadequate infrarenal landing zone, calcifications, or extensive thrombus, the endovascular repair in terms of FEVAR or ch-EVAR could be very challenging. In these patients, the OSR is the preferred method of treatment. Although the suprarenal aortic clamping is correlated with a high risk of cardiac stress and renal ischemia, there are many studies demonstrating a low mortality rate (2.5%) and low permanent dialysis rate (3.7%) [13,14,15]. According to the literature, the 30-day mortality rate by the open and endovascular JAA repair is comparable: OSR—3.4 [12], FEVAR—2.53 [13], ch-EVAR—3.7 [14]. Impairment of renal function was found in 25%, 30%, and 36.4%, respectively. New-onset haemodialysis was required in 9.4%, 10%, and 13.6%, respectively [16].

In our study, we reviewed the outcome of 214 patients who underwent an open surgical repair and observed a mortality rate of 1.9% after OSR. Our results are consistent with the statement in the NICE Guideline [17], that by patients with unruptured complex AAA in whom open repair would be suitable, there is no evidence that the endovascular treatment is associated with benefits in terms of perioperative mortality. When the patients survive the perioperative period, those who have undergone EVAR face double the hazard of death of those whose abdominal aneurysm was repaired in an open operation. In this group of patients, it would be inappropriate to recommend the use of complex EVAR as a standard practice. The NICE committee saw that the OSR is increasingly cost-effective in younger patients who would be more likely to survive the open surgery and experience the long-term survival benefit.

In our cohort of 214 patients, we observed in-hospital mortality rate of 1.9%, which is consistent with the published results in other centres at 1.2–5.2% [2,17,18,19,20,21]. The most common mortality cause in our series was a major cardiac event due to coronary artery disease. As demonstrated in Table 4, 7% (*n* = 15) of the patients underwent a reoperation in the early postoperative period due to wound infection (*n* = 6), bowel ischemia (*n* = 4), mechanical bowel obstruction (*n* = 2), and acute lower limb ischaemia (*n* = 3). With 5.6% (*n* = 12), the pulmonary insufficiency was one of the most encountered postoperative complications. Interestingly, the severity of the respiratory insufficiency was related to the invasiveness of the operation. This correlation was already observed and discussed from other authors [18,19]. Surprisingly, despite the 37.3% (*n* = 80) suprarenal clamping, acute renal failure occurred in only 2.3% (*n* = 5) of our patients, which is a relatively low rate according to the literature [18,19].

According to the NICE guideline, on the basis of a thorough analysis, the OSR is increasingly cost-effective in younger patients, which is consistent with the expectations that younger people will typically be more likely to survive the open surgical procedure and experience the long-term survival benefit. It is known that the aneurysm-related mortality rate and the reintervention rate rise significantly postoperatively by 8 years in comparison with the OSR. These factors lead to the suggestion that the OSR is the more appropriate treatment option in younger patients with low perioperative risk [20]. Our cohort consists of relatively younger patients and therefore low perioperative risk. This could be the reason for the low mortality rate.

## 5. Conclusions

In conclusion, our study demonstrated that the OSR in the era of the endovascular aneurysm repair is safe, effective, and durable in terms of graft integrity and preservation of the renal function, even in patients with increased cardiovascular risk. In our study, COPD is the risk factor for mortality, and we believe that younger patients with long life expectancy and low perioperative risk may benefit more from open repair, especially when OSR is performed in specialised high-volume centres with large expertise.

## Figures and Tables

**Table 1:** Medicare aneurysm scoring system.

Risk Factor	Score
Age > 80 years	11
Age 76–80 years	6
Age 71–75	1
Female	4
ESRD	9
CRI, no dialysis	7
CHF	6
PAD or CBVD	3

ESRD, end-stage renal disease; CRI, chronic renal insufficiency; CHF, congestive heart. failure; PAD, peripheral arterial disease; CBVD, cerebrovascular disease. High risk: >11, moderate risk: 3–11, low risk: <3.

**Table 2:** Baseline characteristics of the study population.

	Number of Patients (*n* = 214)	Percentage (%)
**Demographic characteristics**		
Age (years) mean ± SD	65.5 ± 9.3	
Age < 70 years	147	68.7
Age < 80 years	203	94.9
Male gender, *n* (%)	183	85.5
BMI (mean ± SD)	26.2 ± 4.4	
Aneurysm max. diameter (mean ± SD)	58.2 ± 13.4	
**Medical history, *n* (%)**		
Hypertension	129	60
Previous stroke/TIA	14	6.5
COPD	19	8.9
Coronary artery disease	68	31.8
NYHA III–IV	21	9.8
Current/previous smoker	71	33.2
Atrial fibrillation	18	8.4
CKI	22	10.3
Connective tissue disease	5	2.3
Previose aortic surgery	15	7.0
Malignant disease	25	11.7
Peripheral arterial disease	49	22.9
Diabetes mellitus	17	7.9
**Medication treatment, *n* (%)**		
β-Blocker	93	43.5
ACEIs	63	29.4
Aspirin	107	50
Anticoagulation	28	13.1
Statins	97	45.3

TIA, transient ischemic attack; ACEIs, angiotensin-converting enzyme inhibitors; COPD, chronic obstructive pulmonary disease; CKI, chronic kidney injury; BMI, body mass index; NYHA, New York Heart Association.

**Table 3:** Baseline characteristics and procedural data.

Surgical Risk Patients	Number of Patients (*n* = 214)	Percentage (%)
Low risk	134	62.6
Moderate risk	61	28.5
High risk	19	8.9
Procedure characteristics		
**Aortic access**		
Retroperitoneal	23	10.7
Transperitoneal	182	85
Suprarenal clamping	80	37.3
Reimplantation of IMA	14	6.5
OP duration (minutes), mean (SD)	189.5 ± 78.7	
**ICU stay (days), median (SD)**	6.5 ± 5.4	
Infrareal aneurysm, median (SD)	5.0 ± 0.9	
Juxtarenal aneurysm, median (SD)	60.0 ± 6.1	
**In-hospital stay (days), mean (SD**)	11.0 ± 10.9	
Infrareal aneurysm, median (SD)	10.0 ± 12.4	
Juxtarenal aneurysm, median (SD)	11.0 ± 7.7	
IMA, inferior mesenteric artery		

IMA, inferior mesenteric artery; SD, standard deviation.

**Table 4:** Postoperative outcomes.

Early Complications	Number of Patients (*n* = 214)	Percentage (%)
Need for reoperation	15	7
In-hospital mortality	4	1.9
Wound infection	13	6.1
Lower limb ischemia	6	2.8
Pulmonary complication	12	5.6
Myocardial infarction	3	1.4
Bowel ischemia	4	1.9
Ileus	12	5.6
Urinary tract infection	9	4.2
Bleeding (re-operation)	3	1.4
**Late Complications**	**Number of Patients**	**Percentage (%)**
Incisional hernia	16	7.5
Kidney failure	5	2.3
Anastomosis aneurysm	2	0.9
Prosthesis infection/AEF	4	1.9
Vascular prosthesis obstruction	1	0.5
Death at the end of FU	12	5.6

AEF, aortoenteric fistula; FU, follow-up.

**Table 5:** Multivariate analysis.

	Univariable Analysis			Multivariate Logistic Regression		
	OR	95% CI	*p*-Value	OR	95% CI	*p*-Value
High risk factor (MASS)	4.1	1.1–15.8	**0.034**	2.1	0.38–11.37	0.388
COPD	31.8	3.1–324	**0.003**	23.4	1.8–295	**0.015**
Transfusion of PRBCs	2.07	1.33–3.2	**0.001**	1.6	0.85–3.21	0.139

CI: confidence interval; OR: odds ratio; MASS, Medicare Aneurysm Scoring System; COPD, chronic obstructive pulmonary disease; PRBC, Packed red blood cells. Statistically significant *p*-values are marked in bold.

## Data Availability

Not applicable.
